# Alveolar Echinococcosis, Lithuania

**DOI:** 10.3201/eid1310.061161

**Published:** 2007-10

**Authors:** Rasa Bružinskaitė, Audronė Marcinkutė, Kęstutis Strupas, Vitalijus Sokolovas, Peter Deplazes, Alexander Mathis, Carlos Eddi, Mindaugas Šarkūnas

**Affiliations:** * Lithuanian Veterinary Academy, Kaunas, Lithuania; † University Hospital of Tuberculosis and Infectious Diseases, Vilnius, Lithuania; ‡ Santariškių Clinic, Vilnius, Lithuania; § Institute of Parasitology, Zürich, Switzerland; # Food and Agriculture Organization, Rome, Italy

**Keywords:** Echinococcosis, hepatic alveolar, Echinococcus multilocularis, fox, Lithuania, letter

## Abstract

Alveolar Echniococcosis, Lithuania

**To the Editor:** Alveolar echinococcosis (AE), a serious zoonosis caused by the tapeworm *Echinococcus multilocularis*, has been reported in neighboring countries of Lithuania in recent years (*1*–*4*), but no published epidemiologic information is available. The red fox (*Vulpes vulpes*), the main definitive host of *E. multilocularis* in Europe (*1*), and important intermediate rodent hosts (e.g. *Arvicola terrestris, Microtus arvalis*) are present in Lithuania (*5*), but to date they have not been investigated systematically. The helminth fauna of carnivores in Lithuania had been investigated in a study in 1976, but no record was made for *E. multilocularis* (*6*). Notably, *E. multilocularis* has recently been identified in 1 of 5 muskrats (*Ondatra zibethicus*) captured in the Šilutė district of Lithuania (*7*). The objectives of our study were to estimate the prevalence of *E. multilocularis* in definitive hosts and to gather first information concerning AE in humans in Lithuania.

From 1997 to July 2006, 80 AE cases have been diagnosed at the reference hospital for AE, the Hospital of Tuberculosis and Infectious Diseases in cooperation with the Santariškių Clinic, Vilnius University. Diagnoses were based on serologic testing using ELISA (Bordier Affinity, Crissier, Switzerland) and Western blot (LDBIO, Lyon, France) or imaging methods (ultrasound scan, computed tomography). In 6.7% of the cases identified by imaging techniques, serum antibodies were not detected by ELISA. Diagnoses in all cases were confirmed by histopathologic examination or typical liver lesion morphologic features. Most of the cases were registered in the past 5 years (10–16 cases/year in 2002–July 2006 compared with 0–4 cases/year in 1997–2001). In 26 (33%) of 80 patients, metacestodes were found in the bilateral liver lobes; in 20 (25%) metacestodes were found in the right lobe. Metacestodes had also spread into extra hepatic tissues and metastasized to the right lung, right kidney, spleen, and genitals in 18 (23%) of the patients. AE was diagnosed in 62 (78%) of patients in the third to fourth clinical stage of the disease, according to the PNM (P, parasitic mass in the liver; N, involvement of neighboring organs; M, metastasis) classification: P2-3N0-1M0, P4N1M1 (*8*); twelve (15%) patients died, 7 of them within 4–24 months after diagnosis. The patients’ ages varied from 21 to 83 years (mean age 58 years). Women were more frequently infected (63%) than men (38%), which could be explained by women’s more frequent involvement in gardening. Eighty-one percent of AE patients were farmers or persons involved in agricultural activities. Most AE patients originated in the northwestern and northeastern parts of Lithuania, but cases were recorded from many parts of the country (Figure), which suggests that the whole territory of Lithuania should be considered as an AE-endemic area.

**Figure:**
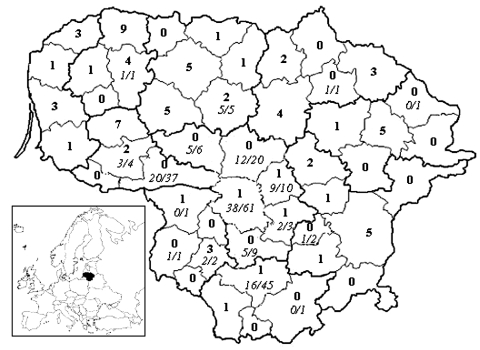
Number of patients (shown in **boldface**) diagnosed with human alveolar echinococcosis at the Hospital of Tuberculosis and Infectious Diseases, Vilnius University, from 1997 through July 2006 in districts of Lithuania. No. *Echinococcus multilocularis–*positive/no. red foxes (*Vulpes vulpes*) (shown in *italics*) investigated during 2001–2004 is indicated for some of the districts.

To assess the prevalence of *E. multilocularis* in definitive hosts, the small intestines of 206 hunted red foxes were collected from randomly selected districts from October 2001 to April 2004 and examined following strict safety precautions by the sedimentation and counting technique. *E. multilocularis* was detected in 118 red foxes (57.3%, 95% confidence interval [CI] 50.2%–64.1%). The tapeworm was present in foxes from most tested localities; the highest prevalence of 62.3% (CI 49.0–74.4%) was observed in the Kaunas district (Figure). The median worm burden per infected fox was 56 (1–20, 924) in this district. The high prevalences of *E. multilocularis* in foxes in the examined areas support the hypothesis that foxes play the key role as definitive hosts in the biology of this tapeworm in Lithuania.

In the framework of an epidemiologic investigation on *E. granulosus,* the contents of small necrotic lesions (size 3–8 mm) found in 21 randomly collected pig livers from small family farms in the southwestern part of Lithuania were also investigated by PCR (*9*); 3 lesions were positive for AE. Further, 2 of 34 dogs from rural areas in the southwestern part of Lithuania shed taeniid eggs in feces that were positive for *E. multilocularis* on examination with a multiplex PCR (*10*).

The high number of human AE cases and the high prevalence of *E. multilocularis* in definitive wild hosts document that AE is of emerging concern in Lithuania. However, this study cannot conclusively document a recent extension of the parasite’s range and an increase of the infection pressure. Clearly, the identification of AE in pigs and of *E. multilocularis* in dogs from small family farms demonstrates that transmission of *E. multilocularis* occurs in rural environments in close proximity to the population.
